# The risk of hemochromatosis among first- and second-generation immigrants: a cohort study of the total population in Sweden

**DOI:** 10.48101/ujms.v129.10376

**Published:** 2024-08-09

**Authors:** Per Wändell, Xinjun Li, Axel C. Carlsson, Jan Sundquist, Kristina Sundquist

**Affiliations:** a Division of Family Medicine and Primary Care, Department of Neurobiology, Care Sciences and Society, Karolinska Institutet, Huddinge, Sweden; b Center for Primary Health Care Research, Lund University, Malmö, Sweden; c Academic Primary Health Care Centre, Stockholm Region, Stockholm, Sweden; d University Clinic Primary Care Skåne, Region Skåne, Sweden; e Center for Community-based Healthcare Research and Education (CoHRE), Department of Functional Pathology, School of Medicine, Shimane University, Matsue, Japan

**Keywords:** Hemochromatosis, immigrants, neighborhood, sex, socioeconomic status

## Abstract

**Purpose:**

We aimed to analyze the risk of hereditary hemochromatosis (HH) among first-generation and second-generation immigrants in Sweden using Swedish-born individuals and Swedish-born individuals with Swedish-born parents as referents, respectively.

**Methods:**

All individuals aged 18 years of age and older, *n* = 6,180,500 in the first-generation study, and *n* = 4,589,930 in the second-generation study were included in the analyses. HH was defined as at least one registered diagnosis International Classification of Diseases 10th edition (E83.1) in the National Patient Register between January 1, 1998 and December 31, 2018. Cox regression was used to estimate the hazard ratios (HRs) with 99% confidence intervals (CI) owing to multiple testing, of incident HH with adjustments for age, cancer, other comorbidities, and socio-demographics.

**Results:**

In the first-generation study, there were 5,112 cases of HH, and in the second-generation study 4,626 cases of HH. The adjusted HRs for first-generation men and women overall were 0.72 (99% CI: 0.63–0.82) and 0.61 (99% CI: 0.52–0.72), respectively, and for the second-generation men and women 0.72 (99% CI: 0.62–0.83) and 0.97 (99% CI: 0.83–1.14), respectively, with a higher risk found only among first-generation men from Western Europe, HR 1.47 (99% CI: 1.05–2.06), compared to the control group.

**Conclusions:**

Our findings indicate that the overall risk of HH was lower among both first-generation and second-generation immigrants when compared to individuals born in Sweden or with Swedish-born parents. An elevated risk for HH was observed exclusively among first-generation men originating from Western Europe. These findings represent new knowledge and should be of global interest.

## Background

Hereditary hemochromatosis (HH) is a disease that affects many different organs, with clinical manifestations including liver cirrhosis and carcinoma, cardiomyopathy, arthropathy, diabetes and hypogonadism, also called ‘bronze diabetes’ due to the darkening of the skin and hyperglycemia (1). HH is an autosomal recessive disorder characterized by iron overload and is more common in individuals of Northern and Western European descent (2). Ethnic differences have been described to be associated with HH, with higher Tyr allele frequency in native Swedes and lower frequencies in Asiatic populations and ‘the fact that this allele appears to be rare or absent in Indians and Chinese is in agreement with previous observations that hemochromatosis is found mainly in Europe and in populations of European origin’ (3). Males are more commonly affected by HH than females (4).

HH has been associated with other concomitant diseases, such as cardiomyopathies (5), squamous cell carcinoma in the esophagus, and adenocarcinoma in the colon (6).

We expected a lower risk among immigrants to Sweden from regions outside of Europe. However, the magnitude of the difference is not known and the risk in some immigrant groups is unknown. Sweden, with a large immigrant population with different origins and reliable national registers, ought to be a suitable population to study these differences, which should be of global interest. The aim of this study was to investigate the risk of HH in first- and second-generation immigrants in Sweden.

## Methods

### Design

We used national Swedish registers using a retrospective register data design. The following nationwide registers were used: the Swedish Total Population Register (TPR), and the Swedish National Patient Register (NPR), that is, registers from Statistics Sweden and the National Board of Health and Welfare. The TPR includes data on births, deaths, marital status, family relationships, and migration, both within and to/from Sweden. The completeness of the TPR is high, with the inclusion of 100% of births and deaths, 95% of immigrations, and 91% of emigrations (7). The NPR includes International Classification of Diseases 10th edition (ICD-10) diagnoses from all of Sweden since 1997, with more than 99% of all somatic and psychiatric hospital discharges being registered (8). For outpatient hospital care, diagnoses are included nationwide from 2001 specialist care, but not from primary health care, and the rate of missing data is estimated to be 3% (9). The follow-up period ran from January 1, 1998 until hospitalization/out-patient treatment of HH, death, emigration, or the end of the study period on December 31, 2018, whichever came first.

### Population

The total population of individuals 18 years of age and older in Sweden were included. Immigrants were defined as individuals residing in Sweden with a foreign origin. We included first-generation immigrants, that is, foreign-born individuals, with Swedish-born individuals as referents, and second-generation immigrants, that is, Swedish-born individuals with at least one foreign-born parent, with Swedish-born individuals with two Swedish-born parents as referents.

### Outcomes

Hereditary hemochromatosis was defined as the ICD10-code E83.1.

### Comorbidities

For comorbidities, we included the following diagnoses (ICD-10 codes): diabetes mellitus (E10-E14), hypertension: (I10-I19), coronary heart disease (CHD; I20-I25), chronic rheumatic heart diseases (I05-I09), non-rheumatic valvular heart diseases (I34-I39), cardiomyopathy (I42-I43), atrial fibrillation (AF; I48), stroke (I60-I69), arthropathy in hemochromatosis (M14.5), cancers in gastrointestinal organs (C15-C21), and other cancers outside the gastrointestinal system (C00-C14, C22-C97).

### Demographic and socioeconomic variables

The study population was stratified by sex, and age was used as a continuous variable, in the analysis.

Educational attainment was categorized as: ≤ 9 years (partial or complete compulsory schooling), 10–12 years (partial or complete secondary schooling) and > 12 years (attendance at college and/or university).

#### Marital status was set as married or not

Geographic region of residence was included to adjust for possible regional differences in disease incidence, and regional differences in hospital admissions/specialist care visits and was categorized as (1) large cities, (2) southern Sweden and (3) northern Sweden. Large cities were defined as municipalities with a population of > 200,000 and comprised the three largest cities in Sweden: Stockholm, Gothenburg, and Malmö. Southern Sweden includes the regions of Götaland and Svealand, and northern Sweden the region of Norrland. The boundary is at the river of Dalälven.

### Neighborhood socioeconomic status

Neighborhood socioeconomic status (NSES) was derived from Small Area Market Statistics (SAMS) data. SAMS were originally created for commercial purposes and are registered as small geographic areas, with borders defined by homogenous types of buildings. The average population in each SAMS neighborhood is approximately 2,000 people in the Stockholm area, and 1,000 people in the rest of Sweden. A summary index was calculated to characterize neighborhood-level SES and was based on four variables: low educational status (<10 years of formal education); low income (including income from all sources, including that from interest and dividends, defined as less than 50% of individual median income); unemployment (defined as not employed, excluding full-time students, those completing compulsory military service, and early retirees); and social welfare payment (receiving social welfare support) (10). This summary index was categorized into four groups: more than one standard deviation (SD) below the mean (high SES), more than one SD above the mean (low SES), and within one SD of the mean (moderate SES). The group with high SES was used as the reference group. Unknown neighborhood SES comprised its own group.

### Statistical analysis

Baseline data are presented with categorical variables as counts and percentages. We used Cox regression analysis to estimate hazard ratios (HR) with 99% confidence intervals (CI) (owing to multiple testing), of newly diagnosed HH in different groups of immigrants compared to Swedish-born individuals during the follow-up time. We tested the proportional hazard assumptions by plotting the incidence rates over time and by calculating Schoenfeld (partial) residuals and these assumptions were fulfilled. All analyses were stratified by sex. Three models were used: Model 1 with adjustment for age and region of residence in Sweden; Model 2 with adjustment for age, region of residence, educational level, marital status, and neighborhood SES; and Model 3 as Model 2 but with the inclusion of the comorbidities. Regions with less than 10 cases are not shown in the main Tables. We also performed interaction tests to evaluate whether the association between immigrant status and HH risk varied according to region of Sweden or neighborhood SES. The calculations were performed using the statistical program SAS.

## Results

In total, 6,180,500 individuals were included in the first-generation study, with 5,112 cases of HH, and 4,589,930 were included in the second-generation study, with 4,626 cases (Supplementary Table 1). In general, more men than women had HH, both among Swedish-born and foreign-born.

In the first-generation study, more Swedish-born men and women with higher education were affected by HH (Tables 1 and 2), although no statistically significant differences among the women with the highest education were seen. Among foreign-born, no statistically significant differences were found. Regarding region in Sweden and among Swedish-born, there were fewer cases in southern Sweden and more cases in northern Sweden among both men and women, while among foreign-born men but not among foreign-born men women, there were fewer cases both in southern and northern Sweden, compared to the three largest cities. Among Swedish-born, there were more married men and women affected by HH. Regarding neighborhood SES, there were fewer cases among men and women in the highly deprived areas, especially among Swedish-born. For comorbidities, more Swedish-born men and foreign-born men and women were affected by diabetes, more Swedish-born men and women were affected by hypertension, cardiomyopathy, and cancers besides gastrointestinal cancers, and among men also AF, while CHD and stroke were less common among Swedish-born men. As regards arthropathy in HH, this was more common in both Swedish- and foreign-born men and women. Among foreign-born men and women, cancers besides gastrointestinal cancers were more common, also hypertension among men and AF among women.

**Table 1:** The hazard ratios (HRs) of hemochromatosis among first-generation Swedish-born men and foreign-born immigrant men expressed as HR with 95% confidence intervals (95% CI).

	Swedish-born			Foreign-born		
	HR*	95% CI		HR*	95% CI	
Birth year	1.00	1.00	1.00	1.01	1.00	1.02
Educational level (ref. ≤ 9 year)						
10–12	1.12	1.02	1.23	0.84	0.62	1.15
> 12	1.15	1.03	1.28	0.93	0.67	1.31
Region of residence (ref. Large cities)						
Southern Sweden	0.72	0.66	0.80	0.69	0.50	0.95
Northern Sweden	1.59	1.45	1.74	0.39	0.20	0.76
Marital status (ref. Not married)	1.22	1.11	1.33	0.85	0.65	1.13
Neighborhood deprivation (ref. Low)						
Middle	0.85	0.78	0.93	0.70	0.50	0.96
High	0.76	0.66	0.87	0.51	0.35	0.73
Unknown	0.28	0.19	0.43	0.60	0.27	1.37
**Comorbidities (ref. Non)**						
Diagnosis of diabetes	1.71	1.53	1.91	1.22	0.85	1.74
Diagnosis of hypertension (ref. Non)	1.64	1.50	1.80	2.29	1.69	3.11
Diagnosis of coronary heart disease	0.76	0.66	0.86	0.72	0.48	1.08
Diagnosis of chronic rheumatic heart disease	1.31	0.62	2.79			
Diagnosis of non-rheumatic valvular heart disease	1.03	0.84	1.27	1.46	0.74	2.88
Diagnosis of cardiomyopathy	1.44	1.03	2.02	0.83	0.20	3.40
Diagnosis of atrial fibrillation	1.48	1.28	1.71	1.53	0.93	2.51
Diagnosis of stroke	0.81	0.70	0.93	0.91	0.58	1.45
Cancers in gastrointestinal organs	0.85	0.68	1.08	0.95	0.46	1.95
Other cancers	1.47	1.34	1.62	2.26	1.66	3.09

* Fully adjusted.

**Table 2:** The hazard ratios (HRs) of hemochromatosis among first-generation Swedish-born women and foreign-born immigrant women expressed as HR with 95% confidence intervals (95% CI).

	Swedish-born			Foreign-born		
	HR*	95% CI		HR*	95% CI	
Birth year	1.00	0.99	1.00	0.98	0.97	1.00
Educational level (ref. ≤ 9 year)						
10–12	1.30	1.16	1.45	1.11	0.76	1.61
> 12	1.15	1.01	1.31	0.89	0.56	1.40
Region of residence (ref. Large cities)						
Southern Sweden	0.67	0.60	0.75	0.74	0.49	1.12
Northern Sweden	1.62	1.46	1.80	1.41	0.88	2.26
Marital status (ref. Not married)	1.29	1.17	1.41	1.22	0.87	1.71
Neighborhood deprivation (ref. Low)						
Middle	0.80	0.72	0.89	0.84	0.56	1.25
High	0.77	0.66	0.90	0.48	0.30	0.79
Unknown	0.18	0.09	0.36	0.15	0.06	0.34
**Comorbidities (ref. Non)**						
Diagnosis of diabetes	1.54	1.33	1.78	2.09	1.35	3.25
Diagnosis of hypertension (ref. Non)	1.58	1.42	1.76	1.22	0.83	1.79
Diagnosis of coronary heart disease	0.93	0.79	1.09	0.87	0.49	1.56
Diagnosis of chronic rheumatic heart disease	1.15	0.51	2.60			
Diagnosis of non-rheumatic valvular heart disease	1.12	0.87	1.45	0.90	0.34	2.36
Diagnosis of cardiomyopathy	2.26	1.51	3.39	1.07	0.15	7.81
Diagnosis of atrial fibrillation	1.14	0.94	1.38	2.03	1.07	3.83
Diagnosis of stroke	0.88	0.74	1.04	0.54	0.27	1.08
Cancers in gastrointestinal organs	1.16	0.90	1.49	0.19	0.03	1.35
Other cancers	1.67	1.50	1.85	1.79	1.23	2.61

* Fully adjusted.

In the second-generation study (Table 3), HR for men versus women with Swedish-born parents was significantly higher (HR 1.46, 99% CI: 1.37–1.55) but not so for men versus women with foreign-born parents (HR 1.19, 99% CI: 0.97–1.46). For individuals with Swedish-born parents, fewer were affected in southern Sweden and more in northern Sweden compared to the three largest cities, while among individuals with foreign-born parents, this was true only for southern Sweden. Regarding neighborhood SES, there were fewer affected by HH among individuals in the highly deprived areas, especially among those with Swedish-born parents. Regarding comorbidities, these were more common in both individuals with Swedish- and foreign-born parents for diabetes, hypertension, arthropathy in HH and cancers besides gastrointestinal cancers. In addition, cardiomyopathy and AF were more common in individuals with Swedish-born parents, and CHD was less common.

**Table 3:** The hazard ratios (HRs) of hemochromatosis among second-generation Swedish-born and foreign-born immigrants expressed as HR with 95% confidence intervals (95% CI).

	Swedish-born			Foreign-born		
	HR*	95% CI		HR*	95% CI	
Birth year	0.98	0.98	0.99	0.97	0.96	0.98
Gender to males (ref. Females)	1.46	1.37	1.55	1.19	0.97	1.46
Educational level (ref. ≤ 9 year)						
10–12	1.17	1.08	1.26	1.26	0.96	1.66
> 12	1.12	1.03	1.22	1.13	0.83	1.55
Region of residence (ref. Large cities)						
Southern Sweden	0.70	0.65	0.76	0.75	0.58	0.96
Northern Sweden	1.69	1.57	1.81	0.85	0.62	1.16
Neighborhood deprivation (ref. Low)						
Middle	0.83	0.78	0.89	0.89	0.70	1.13
High	0.77	0.69	0.86	0.69	0.49	0.96
Unknown	0.74	0.24	2.31	0.84	0.11	6.23
Diagnosis of diabetes	1.70	1.55	1.86	1.79	1.29	2.50
Diagnosis of hypertension (ref. Non)	1.56	1.45	1.68	1.80	1.38	2.36
Diagnosis of coronary heart disease	0.71	0.63	0.79	0.82	0.55	1.23
Diagnosis of chronic rheumatic heart disease	0.99	0.49	1.99	1.72	0.23	12.93
Diagnosis of non-rheumatic valvular heart disease	1.06	0.89	1.26	1.16	0.58	2.32
Diagnosis of cardiomyopathy	1.33	1.00	1.75	1.81	0.74	4.43
Diagnosis of atrial fibrillation	1.32	1.19	1.46	1.02	0.66	1.57
Diagnosis of stroke	0.87	0.76	0.98	1.18	0.76	1.84
Cancers in gastrointestinal organs	1.00	0.83	1.21	0.40	0.13	1.25
Other cancers	1.51	1.41	1.63	1.76	1.34	2.31

* Fully adjusted.

In the first-generation study, HH was less common among both foreign-born men and women (Table 4). Among both women and men, HH was less common among men and women from Nordic countries, Eastern Europe and Asia. Among foreign-born men from Western Europe, the HRs in models 2 and 3 were significantly higher at 1.47 (99% CI: 1.05–2.06). Regions with less than 10 cases are not presented in the main tables.

**Table 4:** The hazard ratios (HRs) of hemochromatosis in first-generation male and female immigrants versus Swedish-born men expressed as HR with 99% confidence intervals (99% CI); regions with < 10 individuals are excluded within this table.

	Cases	Model 1			Model 2			Model 3		
		HR	99% CI		HR	99% CI		HR	99% CI	
Males										
Sweden	2,689	1	-	-	1	-	-	1	-	-
All foreign-born	267	**0.54**	**0.48**	**0.61**	**0.72**	**0.63**	**0.83**	**0.72**	**0.63**	**0.82**
Nordic countries	88	**0.56**	**0.45**	**0.69**	**0.77**	**0.62**	**0.96**	**0.78**	**0.62**	**0.96**
Southern Europe	13	**0.45**	**0.26**	**0.78**	0.69	0.40	1.19	0.71	0.41	1.23
Western Europe	35	1.13	0.81	1.58	**1.48**	**1.06**	**2.07**	**1.47**	**1.05**	**2.06**
Eastern Europe	23	**0.36**	**0.24**	**0.54**	**0.44**	**0.29**	**0.66**	**0.44**	**0.29**	**0.67**
Central Europe	20	0.79	0.51	1.23	0.98	0.63	1.52	0.96	0.62	1.50
Africa	13	**0.47**	**0.27**	**0.82**	0.65	0.38	1.12	0.64	0.37	1.10
North America	10	1.03	0.55	1.92	1.52	0.81	2.83	1.58	0.85	2.96
Asia	51	**0.43**	**0.33**	**0.57**	**0.57**	**0.43**	**0.76**	**0.58**	**0.43**	**0.77**
Females										
Sweden	1,996	1	-	-	1	-	-	1	-	-
All foreign-born	160	**0.47**	**0.40**	**0.55**	**0.62**	**0.52**	**0.73**	**0.61**	**0.52**	**0.72**
Nordic countries	84	**0.61**	**0.49**	**0.76**	**0.77**	**0.62**	**0.96**	**0.75**	**0.60**	**0.94**
Western Europe	12	0.58	0.33	1.02	0.72	0.41	1.27	0.70	0.40	1.24
Central Europe	12	**0.56**	**0.32**	**0.99**	0.66	0.37	1.16	0.66	0.37	1.16
Asia	20	**0.30**	**0.19**	**0.46**	**0.41**	**0.26**	**0.64**	**0.41**	**0.26**	**0.64**

Model 1: adjusted for age; model 2: adjusted for age, region of residence in Sweden, educational level, and marital status, and neighborhood deprivation; model 3: model 2 + comorbidities. Bold values are statistically significant.

Lower risks were seen for several groups, such as men from Nordic, Baltic, and Asian Countries in the second-generation study of men (Table 5). In the second-generation study of women, there was only one statistically significant HR, that is, a lower risk among individuals with parents from Eastern Europe. Regions with less than 10 cases are not presented in the main tables.

**Table 5:** The hazard ratios (HRs) of hemochromatosis in second-generation male and female immigrants versus Swedish-born men expressed as HR with 99% confidence intervals (99% CI).

	Cases	Model 1			Model 2			Model 3		
		HR	99% CI		HR	99% CI		HR	99% CI	
Males										
Sweden	2,561	1	-	-	1	-	-	1	-	-
All with foreign-born parents	208	**0.73**	**0.63**	**0.84**	**0.73**	**0.64**	**0.85**	**0.72**	**0.62**	**0.83**
Nordic countries	129	**0.78**	**0.65**	**0.93**	**0.77**	**0.64**	**0.92**	**0.78**	**0.65**	**0.93**
Southern Europe	10	0.98	0.53	1.83	1.01	0.54	1.88	1.03	0.55	1.91
Western Europe	25	0.76	0.51	1.13	0.76	0.51	1.14	0.78	0.52	1.16
Central Europe	12	**0.84**	**0.48**	**1.48**	0.87	0.49	1.54	0.81	0.46	1.44
North America	11	1.25	0.69	2.26	1.24	0.69	2.25	1.19	0.66	2.16
Females										
Sweden	1,695	1	-	-	1	-	-	1	-	-
All with foreign-born parents	162	0.95	0.81	1.12	0.97	0.82	1.14	0.97	0.83	1.14
Nordic countries	116	1.15	0.95	1.39	1.13	0.94	1.37	1.13	0.94	1.37
Western Europe	14	0.74	0.44	1.25	0.77	0.45	1.30	0.79	0.46	1.33

Model 1: adjusted for age; model 2: adjusted for age, region of residence in Sweden, educational level, and marital status, and neighborhood deprivation; model 3: model 2 + comorbidities. Bold values are statistically significant.

An interaction test was performed to evaluate whether the association between immigrant status and HH risk varied according to region of Sweden or neighborhood SES, there were significant interactions between immigrant status and region of residence and neighborhood SES among first-generation men and immigrant status and region of residence in the second-generation men and women. For example, there were higher risks of HH for the first-generation individuals and the second-generation individuals with both Swedish born parents lived in northern Sweden.

## Discussion

The main results from this study were that, in general, foreign-born men and women showed a lower risk of being diagnosed with HH, with a higher risk only seen in men from Western Europe. Among second-generation individuals, that is, Swedish-born but with foreign-born parents, a lower risk was seen in general among men, and in the group with parents from the Nordic countries.

A higher risk of HH is known in some regions of the world, such as in European countries, especially Western and Northern Europe (11), including Sweden (3), and especially northern Sweden (12). Thus, our results with lower risks among most foreign-born but a higher risk among men from Western Europe, are in line with previous findings. The reduced risk of HH observed among immigrants could be attributed to several factors, for example, genetic diversity among populations (2, 3, 12), and geographic variability in HH-associated mutations, dietary and lifestyle differences, cultural practices, variations in health care systems and screening practices, the migration effect, and selective migration (1).

Some findings among background factors indicate that the disease might be under-diagnosed, that is, the higher risk among Swedish-born with higher education, being married, and also the higher risk in less deprived neighborhood areas, where knowledge about the disease could lead to more frequent contact with health care.

Among comorbidities, arthropathies are well-known (11). Cardiovascular diseases are also known to be more common in individuals with HH, such as ischemic heart disease (5), while we found this to be less common. Instead, we found a higher risk of cardiomyopathy (5), and to some extent also of AF. Diabetes is also a common comorbidity but was not significantly higher among foreign-born men.

Regarding gastrointestinal cancers, we found this to be less common in contrast to earlier findings (6) but, on the other hand, other cancers outside the gastrointestinal system were more frequent. However, only some specific gastrointestinal cancer forms have been associated with HH, that is, squamous cell carcinoma in the esophagus, and adenocarcinoma in the colon (6).

There are some limitations of this study. We used nationwide register-based data with no possibility to check detailed clinical data, which means that we were unable to assess the methods for diagnosis or the mechanisms behind our findings. In general, Swedish registers are found to be of high quality and validity (7, 8). In the NPR, diagnoses from primary care are not included, but we presume that possible cases should be referred to hospitals specialists. However, there might be an underdiagnosis of the condition.

In summary, our findings indicate that the overall risk of HH was lower among both first-generation and second-generation immigrants when compared to individuals born in Sweden or those with Swedish-born parents. After adjusting for age, cancer, other comorbidities, and sociodemographic factors, the risk of HH remained notably reduced among first-generation immigrants from various geographical regions. A slightly elevated risk for HH was observed exclusively among first-generation men originating from Western Europe. This study offers valuable insights into the HH risk profiles across different immigrant groups and provides pertinent information for health care policies and clinical practice. It suggests the existence of genetic and environmental factors influencing HH risk, which may vary among distinct population subgroups. Further research is needed to elucidate these associations and for the development of targeted prevention and screening strategies for HH within diverse societal segments. For clinicians, it is important to be aware of the increased risk of HH in certain immigrant groups where this study showed a higher risk of HH in men from Western Europe. In clinical settings, testing high-risk groups and their relatives is important to identify cases earlier and avoid complications. It may also be important to screen those underserved population groups that rarely seek health care to avoid missing those cases that risk to be undiagnosed.

## Ethics and consent

All procedures performed in studies involving human participants were in accordance with the ethical standards of the institutional and/or national research committee and with the 1964 Helsinki Declaration and its later amendments or comparable ethical standards.

Informed consent was not applicable, as the study was based on pseudonymized data from registers. Research data are not shared.

The study was approved by the Regional Ethical Review Board in Lund (ref. no. 2012/795 and later amendments).

This manuscript contains no individual person’s data and, according to the Ethical Review Board in Lund, data can be published without informed consent from the participants.

## Availability of data and material

The datasets generated and/or analyzed during the current study are not publicly available due to restraints in the Swedish legislation but we are willing to collaborate with access to the databases upon request to the authors (Kristina Sundquist and Jan Sundquist).

## Disclosure statement

The authors have no conflict of interest to report.

## Notes on contributors

PW: concept, manuscript drafting, revisions.

XL: statistical analysis, constructive review of manuscript.

ACC: concept, manuscript drafting and review.

JS: concept, acquisition of data, constructive review of manuscript.

KS: concept, acquisition of data, constructive review of manuscript, funding.

## Supplementary Material


