# Supplementary material for: The risk of hemochromatosis among first- and second-generation immigrants: a cohort study of the total population in Sweden
Source: Ups J Med Sci. 2024 Aug 9;129:10.48101/ujms.v129.10376. doi: 10.48101/ujms.v129.10376 (PMC11385458; doi:10.48101/ujms.v129.10376)
Supplement: Supplementary file 1 [file UJMS-129-10376-s001.pdf]

Supplementary Tables.

Supplementary Table 1. The populations in first-generation and second-generation studies and the number of cases of hemochromatosis in each study.

	First-generation				Second-generation			
	Population		Cases		Population		Cases	
	No.	%	No	%	No.	%	No	%
Total population	6180500		5112		4589930		4626	
Immigrant status*								
Swedish	5129567	83.0	4685	91.6	4056736	88.4	4256	92.0
Foreign-born	1050933	17.0	427	8.4	533194	11.6	370	8.0
Gender								
Males	2974698	48.1	2956	57.8	2347360	51.1	2769	59.9
Females	3205802	51.9	2156	42.2	2242570	48.9	1857	40.1
Age (years)								
18-39	2389816	38.7	1396	27.3	2279588	49.7	1534	33.2
40-49	1042518	16.9	1119	21.9	989913	21.6	1185	25.6
50-59	1067502	17.3	1495	29.2	957815	20.9	1436	31.0
≥ 60	1680664	27.2	1102	21.6	362614	7.9	471	10.2
Educational level								
≤ 9	2342258	37.9	1441	28.2	1125740	24.5	1086	23.5
10-12	2434645	39.4	2365	46.3	2169962	47.3	2249	48.6
> 12	1403597	22.7	1306	25.5	1294228	28.2	1291	27.9
Region of residence								
Large cities	2731058	44.2	2499	48.9	2191176	47.7	2204	47.6
Southern Sweden	1922840	31.1	1208	23.6	1570915	34.2	1101	23.8
Northern Sweden	1526602	24.7	1405	27.5	827839	18.0	1321	28.6
Marital status								
Married	3491435	56.5	3133	61.3	1993498	43.4	2447	52.9
Not married	2689065	43.5	1979	38.7	2596432	56.6	2179	47.1
Neighborhood deprivation								
Low	1340706	21.7	1516	29.7	1156041	25.2	1385	29.9
Middle	3339980	54.0	2892	56.6	2770700	60.4	2701	58.4
High	907842	14.7	619	12.1	650640	14.2	535	11.6
Unknown	591972	9.6	85	1.7	12549	0.3	5	0.1
Diagnosis of diabetes	462748	7.5	720	14.1	289270	6.3	643	13.9
Diagnosis of hypertension	1126311	18.2	1686	33.0	730252	15.9	1404	30.4
Diagnosis of coronary heart disease	1222954	19.8	1291	25.3	311164	6.8	424	9.2
Diagnosis of chronic rheumatic heart disease	11380	0.2	14	0.3	5190	0.1	9	0.2
Diagnosis of non-rheumatic valvular heart disease	151144	2.4	191	3.7	80814	1.8	149	3.2
Diagnosis of cardiomyopathy	36020	0.6	65	1.3	28229	0.6	57	1.2
Diagnosis of atrial fibrillation	524590	8.5	676	13.2	241002	5.3	502	10.9
Diagnosis of stroke	494413	8.0	427	8.4	209506	4.6	303	6.5
Cancers in gastrointestinal organs	147704	2.4	149	2.9	81364	1.8	120	2.6
Other cancers	1054961	17.1	1394	27.3	675592	14.7	1148	24.8

Supplementary Table 2. The hazards ratios (HRs) of hemochromatosis in first-generation male and female immigrants vs Swedish-born men expressed as hazard ratios (HR) with 95% confidence intervals (95% CI)

		Model 1			Model 2			Model 3		
	Obs.	HR	95% CI		HR	95% CI		HR	95% CI	
Males										
Sweden	2689	1			1			1		
All foreign-born	267	**0.54**	**0.48**	**0.61**	**0.72**	**0.63**	**0.83**	**0.72**	**0.63**	**0.82**
Nordic countries	88	**0.56**	**0.45**	**0.69**	**0.77**	**0.62**	**0.96**	**0.78**	**0.62**	**0.96**
Southern Europe	13	**0.45**	**0.26**	**0.78**	0.69	0.40	1.19	0.71	0.41	1.23
Western Europe	35	1.13	0.81	1.58	**1.48**	**1.06**	**2.07**	**1.47**	**1.05**	**2.06**
Eastern Europe	23	**0.36**	**0.24**	**0.54**	**0.44**	**0.29**	**0.66**	**0.44**	**0.29**	**0.67**
Baltic countries	1	0.18	0.03	1.29	0.22	0.03	1.55	0.22	0.03	1.54
Central Europe	20	0.79	0.51	1.23	0.98	0.63	1.52	0.96	0.62	1.50
Africa	13	**0.47**	**0.27**	**0.82**	0.65	0.38	1.12	0.64	0.37	1.10
North America	10	1.03	0.55	1.92	1.52	0.81	2.83	1.58	0.85	2.96
Latin America	8	**0.39**	**0.19**	**0.78**	0.50	0.25	1.00	0.52	0.26	1.04
Asia	51	**0.43**	**0.33**	**0.57**	**0.57**	**0.43**	**0.76**	**0.58**	**0.43**	**0.77**
Russia	4	0.92	0.35	2.45	1.27	0.47	3.38	0.54	0.20	1.47
Females										
Sweden	1996	1			1			1		
All foreign-born	160	**0.47**	**0.40**	**0.55**	**0.62**	**0.52**	**0.73**	**0.61**	**0.52**	**0.72**
Nordic countries	84	**0.61**	**0.49**	**0.76**	**0.77**	**0.62**	**0.96**	**0.75**	**0.60**	**0.94**
Southern Europe	6	0.48	0.21	1.06	0.81	0.36	1.81	0.84	0.37	1.87
Western Europe	12	0.58	0.33	1.02	0.72	0.41	1.27	0.70	0.40	1.24
Eastern Europe	9	**0.24**	**0.12**	**0.46**	**0.30**	**0.16**	**0.58**	**0.30**	**0.15**	**0.58**
Baltic countries	1	0.20	0.03	1.42	0.25	0.04	1.77	0.24	0.03	1.74
Central Europe	12	**0.56**	**0.32**	**0.99**	0.66	0.37	1.16	0.66	0.37	1.16
Africa	0									
North America	4	0.72	0.27	1.92	1.09	0.41	2.92	1.14	0.43	3.04
Latin America	7	0.52	0.25	1.10	0.70	0.33	1.46	0.72	0.34	1.51
Asia	20	**0.30**	**0.19**	**0.46**	**0.41**	**0.26**	**0.64**	**0.41**	**0.26**	**0.64**
Russia	5	0.96	0.40	2.32	1.38	0.57	3.32	1.39	0.58	3.35

Model 1: adjusted for age; model 2: adjusted for age, region of residence in Sweden, educational level, and marital status, and neighborhood deprivation; model 3: model 2 + comorbidities

Supplementary Table 3. The hazards ratios (HRs) of hemochromatosis in second-generation male and female immigrants vs Swedish-born men expressed as hazard ratios (HR) with 95% confidence intervals (95% CI)

		Model 1			Model 2			Model 3		
	Obs.	HR	95% CI		HR	95% CI		HR	95% CI	
Males										
Sweden	2561	1			1			1		
All with foreign-born parents	208	**0.73**	**0.63**	**0.84**	**0.73**	**0.64**	**0.85**	**0.72**	**0.62**	**0.83**
Nordic countries	129	**0.78**	**0.65**	**0.93**	**0.77**	**0.64**	**0.92**	**0.78**	**0.65**	**0.93**
Southern Europe	10	0.98	0.53	1.83	1.01	0.54	1.88	1.03	0.55	1.91
Western Europe	25	0.76	0.51	1.13	0.76	0.51	1.14	0.78	0.52	1.16
Eastern Europe	8	**0.49**	**0.25**	**0.99**	0.55	0.27	1.10	0.55	0.27	1.10
Baltic countries	4	**0.34**	**0.13**	**0.91**	**0.35**	**0.13**	**0.94**	**0.36**	**0.13**	**0.95**
Central Europe	12	**0.84**	**0.48**	**1.48**	0.87	0.49	1.54	0.81	0.46	1.44
Africa	0									
North America	11	1.25	0.69	2.26	1.24	0.69	2.25	1.19	0.66	2.16
Latin America	1	0.24	0.03	1.71	0.26	0.04	1.83	0.26	0.04	1.82
Asia	4	**0.24**	**0.09**	**0.65**	**0.27**	**0.10**	**0.72**	**0.27**	**0.10**	**0.71**
Russia	4	1.14	0.43	3.04	1.17	0.44	3.12	0.43	0.16	1.19
Females										
Sweden	1695	1			1			1		
All with foreign-born parents	162	0.95	0.81	1.12	0.97	0.82	1.14	0.97	0.83	1.14
Nordic countries	116	1.15	0.95	1.39	1.13	0.94	1.37	1.13	0.94	1.37
Southern Europe	6	1.10	0.49	2.46	1.18	0.53	2.62	1.19	0.54	2.67
Western Europe	14	0.74	0.44	1.25	0.77	0.45	1.30	0.79	0.46	1.33
Eastern Europe	1	**0.12**	**0.02**	**0.84**	**0.14**	**0.02**	**0.96**	**0.14**	**0.02**	**0.96**
Baltic countries	4	0.51	0.19	1.36	0.54	0.20	1.44	0.54	0.20	1.45
Central Europe	6	0.70	0.32	1.57	0.75	0.34	1.67	0.76	0.34	1.69
Africa	1	0.74	0.10	5.25	0.78	0.11	5.55	0.77	0.11	5.51
North America	4	0.65	0.25	1.74	0.66	0.25	1.75	0.61	0.23	1.63
Latin America	3	1.39	0.45	4.31	1.54	0.50	4.79	1.55	0.50	4.83
Asia	3	0.36	0.12	1.13	0.42	0.14	1.32	0.42	0.14	1.32
Russia	4	1.64	0.62	4.39	1.72	0.65	4.60	1.74	0.65	4.65

Model 1: adjusted for age; model 2: adjusted for age, region of residence in Sweden, educational level, and marital status, and neighborhood deprivation; model 3: model 2 + comorbidities
